# High-Density Linkage Mapping of Agronomic Trait QTLs in Wheat under Water Deficit Condition using Genotyping by Sequencing (GBS)

**DOI:** 10.3390/plants11192533

**Published:** 2022-09-27

**Authors:** Nayyer Abdollahi Sisi, Nils Stein, Axel Himmelbach, Seyed Abolghasem Mohammadi

**Affiliations:** 1 Department of Plant Breeding, IFZ Research Centre for Biosystems, Land Use and Nutrition, Justus Liebig University, 35392 Giessen, Germany; 2 Department of Plant Breeding and Biotechnology, Faculty of Agriculture, University of Tabriz, Tabriz 51666, Iran; 3 Leibniz Institute of Plant Genetics and Crop Plant Research (IPK), 06466 Seeland, Germany

**Keywords:** genotyping by sequencing, quantitative trait loci, water deficit, wheat, yield

## Abstract

Improvement of grain yield is the ultimate goal for wheat breeding under water-limited environments. In the present study, a high-density linkage map was developed by using genotyping-by-sequencing (GBS) of a recombinant inbred line (RIL) population derived from the cross between Iranian landrace #49 and cultivar Yecora Rojo. The population was evaluated in three locations in Iran during two years under irrigated and water deficit conditions for the agronomic traits grain yield (GY), plant height (PH), spike number per square meter (SM), 1000 kernel weight (TKW), grain number per spike (GNS), spike length (SL), biomass (BIO) and harvest index (HI). A linkage map was constructed using 5831 SNPs assigned to 21 chromosomes, spanning 3642.14 cM of the hexaploid wheat genome with an average marker density of 0.62 (markers/cM). In total, 85 QTLs were identified on 19 chromosomes (all except 5D and 6D) explaining 6.06–19.25% of the traits phenotypic variance. We could identify 20 novel QTLs explaining 8.87–19.18% of phenotypic variance on chromosomes 1A, 1B, 1D, 2B, 3A, 3B, 6A, 6B and 7A. For 35 out of 85 mapped QTLs functionally annotated genes were identified which could be related to a potential role in drought stress.

## 1. Introduction

Drought caused by climate change affects crop production, especially under water-limited environments. Wheat (*Triticum aestivum* L.) is one of the fundamental global crops which are mostly cultivated in dry regions. Here water deficit can cause up to 50% yield reduction in wheat if compared to production under irrigation conditions [1]. In this context breeding for drought tolerance is a major concern globally.

Components of the grain yield in wheat are complex and multigenic with low heritability which are highly affected by environmental conditions, especially drought stress [2]. Moreover, yield and yield components traits like number of spikes, number of grains per spike, and 1000 kernel weight as well as some morphological and physiological traits have been recognized as relevant traits to drought tolerance [3]. To develop drought tolerant varieties it is essential to understand the genetic basis of these complex traits [4], thus identification of quantitative trait loci (QTL) and genes for important agronomical traits such as grain yield and its components is a key component of crop improvement programs [5]. To date, a large number of QTLs associated with yield component traits and grain yield under water deficit condition [1,2,3,6,7,8,9,10,11,12,13,14] and normal condition [15,16,17,18,19,20,21,22,23,24,25,26,27,28,29] have been identified on all 21 wheat chromosomes.

To precisely map QTLs underlying the traits of interest, a high-density linkage map is a prerequisite. Today, single nucleotide polymorphisms (SNPs) are the marker of choice for the development of high-density linkage map in crops such as wheat due to their abundance, genome coverage, stability, efficiency, and cost effectiveness [7,30]. Among SNP genotyping methods Genotyping-by-Sequencing (GBS) has emerged as a robust approach that allows performing SNP detection and genotyping simultaneously [31]. GBS is an easy, flexible, cost effective and highly reproducible approach that has been applied to dissect the genetic bases of grain yield-related traits in wheat. Zhou et al. [32] used GBS and the iSelect 9K assay to construct linkage map (spanning 2934.1 cM) in a wheat doubled-haploid population and identified 45 QTLs associated with six spike traits. Using 327,609 GBS-based SNP markers in a panel of 768 wheat cultivars Pang et al. [33] detected 395 QTLs for 12 agronomic traits in seven environments. Genome wide association study (GWAS) approach using 78,606 GBS SNP markers was performed to identify QTLs associated with agronomic and disease resistance traits in 44,624 wheat lines [34]. They could identify stable QTLs for GY on chromosomes 2A, 3B, 4D and 6A across multiple environments [34]. Additionally, GWAS study was performed using GBS SNP markers in 720 wheat lines, and genomic regions on chromosomes 2B, 3A, 4A, 5B, 7A and 7B associated with grain yield and yield stability across multiple irrigated and water stress conditions were reported [21].

The Iran #49 (hereafter referred to as #49) × Yecora Rojo recombinant inbred line (RIL) population has been previously characterized for root and shoot traits using SSR and retrotransposon-based (IRAP and REMAP) markers [35]. Due to low marker density in the respective linkage map, the observed marker-QTL linkages were still inappropriate for marker-assisted breeding. In the present study, we applied GBS to construct a high-density genetic linkage map and performed drought-related QTL identification in wheat. Our objectives were to: (1) construct a high-density linkage map using GBS-based SNP markers; (2) map QTLs for grain yield and related traits in a wheat RIL population under irrigated and water deficit conditions; and (3) identify potential candidate genes for these QTLs based on their tight genetic/physical linkage and functional annotation.

## 2. Results

### 2.1. Phenotypic Data Analysis

**Figure 1:**
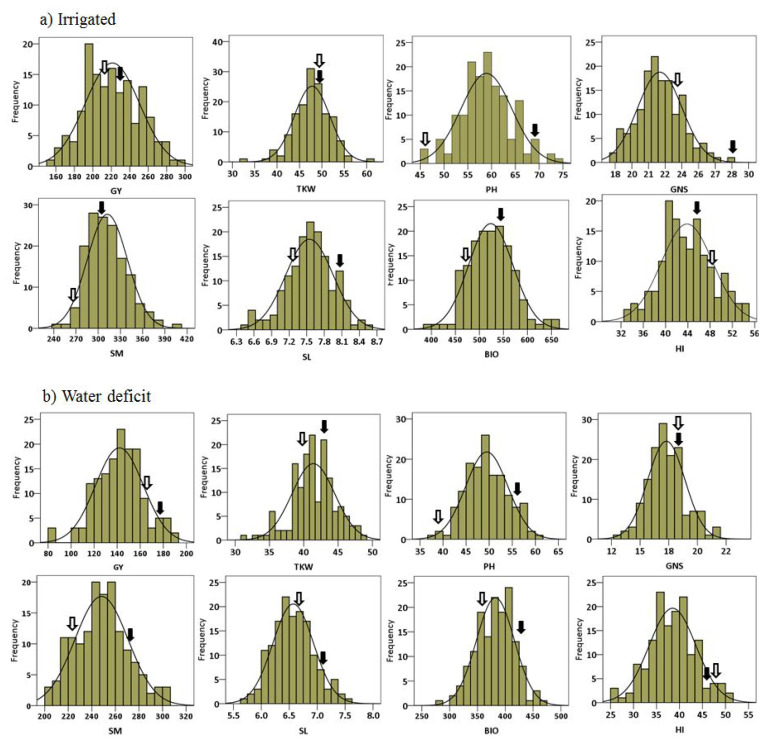
Frequency distribution of averaged phenotypic data of three locations and two years for all traits recorded: under irrigated (**a**); and water deficit (**b**) conditions. #49 and Yecora Rojo are shown by a filled and unfilled arrow, respectively.

A total of 148 RILs as well as parents were grown at three locations and over two years to test performance under irrigation and water deficit regimes. Eight traits were recorded: GY, PH, SM, TKW, GNS, SL, BIO and HI. The combined mean distribution of the traits over six environments (three locations and two years) under irrigated and water deficit conditions are presented in Figure 1a,b, respectively. The results revealed the normal distribution of the traits in the population. For all the studied traits except HI, #49 had higher mean values compared with Yecora Rojo under both conditions (Table S1 supplementary data). GY was positively correlated with all other traits under irrigated and water deficit conditions; however, the correlation between PH and GY under water deficit conditions was not significant. Strong correlations were observed between GY and HI as well as BIO under both conditions. The correlations between SM and PH, SL and GNS were negative and significant under irrigated conditions, whereas these correlations were not significant under water deficit conditions. BIO showed significant positive correlation with all the traits under both conditions except for HI. Negative correlations of SM with GNS, PH, SL and TKW indicate competition between yield components and traits such as plant height for available resources during plant growth (Table 1).

### 2.2. Genome Wide SNP Discovery by GBS

In total, 481.3 million reads were generated and 98% of them were mapped to the Chinese Spring reference genome. The number of reads per samples varied from 2.0 to 4.6 million reads with an average of 3.0 million reads. We required a read depth of four independent sequence reads for homozygous and heterozygous genotype calls, thus our 4-fold coverage SNP matrix was used in subsequent analysis. A total of 35,405 SNPs with a MAF = 0.05 were detected by the SAMtools pipeline based on wheat RefSeqv1.0 [36]. After removing SNPs with ≥ 20% missing values, a total of 7788 polymorphic SNPs distributing across all 21 wheat chromosomes, based on their predicted physical position, were used for further analyses (Table 2). The maximum (785) and minimum (27) number of SNPs were mapped on chromosome 2B and 4D, respectively.

### 2.3. Linkage Map Construction

**Figure 2:**
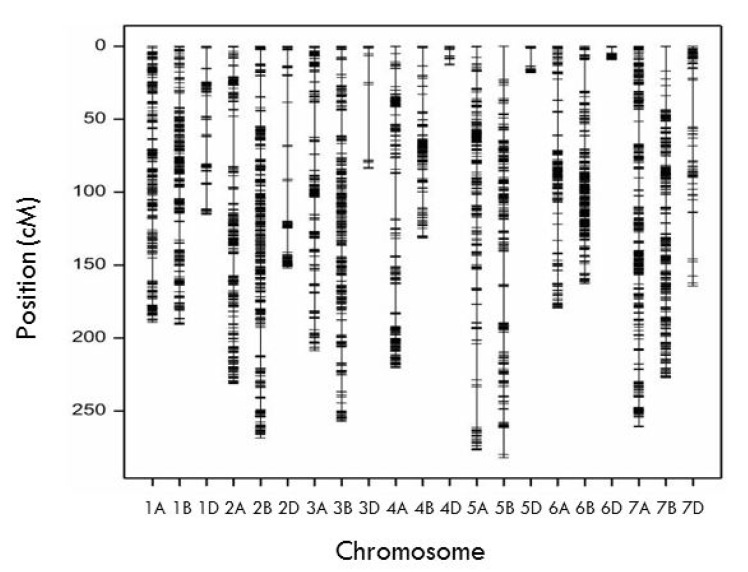
Linkage map constructed from genotyping-by-sequencing in a RIL population derived from a cross between #49 and Yecora Rojo. The genetic distances (cM) of markers are shown on the left side.

A total of 7788 SNP markers were used for map construction and out of which 5831 SNPs were mapped on 21 linkage groups corresponding to the 21 chromosomes of hexaploid wheat (Figure 2). The linkage map spanned a total length of 3642.14 centiMorgan (cM) with an average marker density of 0.62 cM. A total of 2933 (50%) and 2454 (42%) markers were mapped to the B and A genome, respectively, covering 1519.70 and 1566.64 cM of these two genomes. Only 444 out of 5831 SNPs were mapped to the D genome spanning a length of 555.8 cM, indicating a much lower level of polymorphism in D compared to the A and B genomes. The number of markers in the different chromosomes ranged from 570 (2B) to 15 (4D), respectively, and chromosome 3D exhibited the lowest marker density (2.89 cM between two adjacent markers) (Table 2).

### 2.4. QTL Mapping

**Figure 3:**
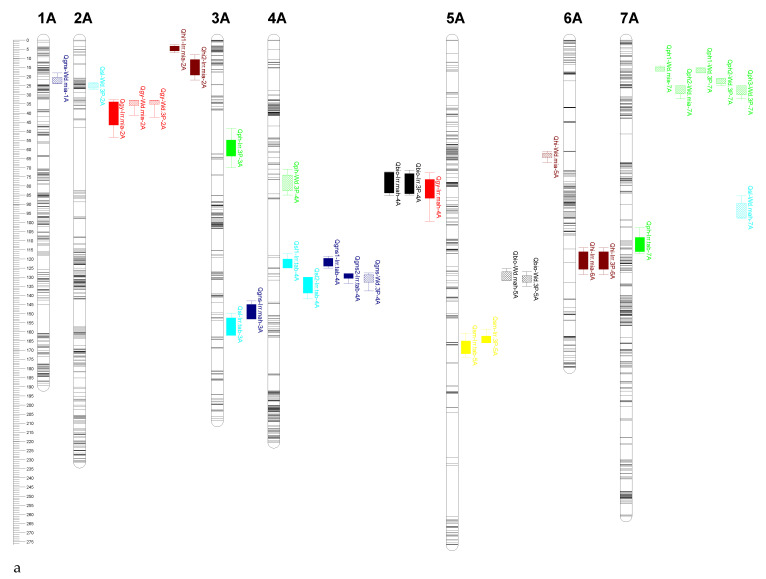

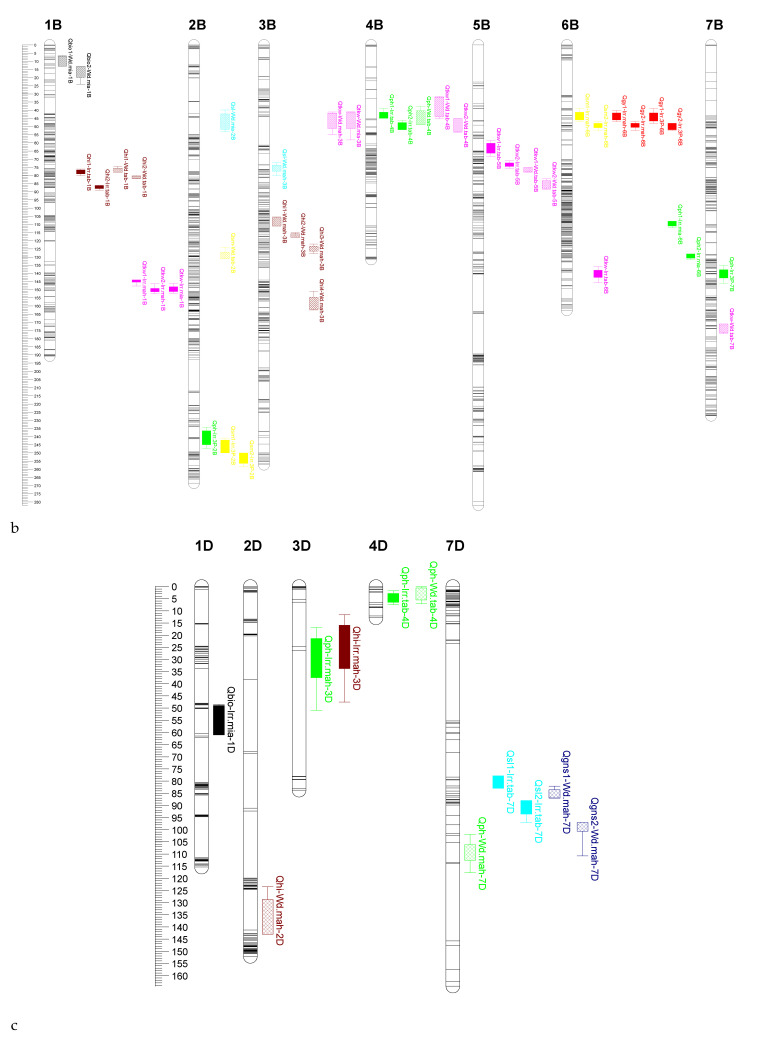
Location of QTLs on the genetic linkage map of wheat developed from the cross #49 × Yecora Rojo under irrigated (solid filled box) and water deficit (stripes filled box) conditions in (**a**) A genome, (**b**) B genome and (**c**) D genome. Each chromosome is represented by map positions by cM (on the left) and identified QTLs (on the right). QTL names indicate the trait, trail (water condition + environment) and chromosome name along with different colors: GY (red), PH (green), SM (yellow), TKW (pink), GNS (blue), SL (light blue), BIO (black), HI (brown).

A total of 85 QTLs were identified for the studied traits across three locations (Mahabad, Miandoab and Tabriz, Iran) during two years and mean of three locations under irrigated and water deficit conditions (Table 3). The Logarithm of Odds (LOD) score of the detected QTLs varied from 2.55 to 10.60 with an average 4.5 explaining 6.06 to 19.25% of the trait’s phenotypic variation (mean 10.6%). No QTL was identified on chromosomes 5D and 6D. The number of QTL varied from 19 for PH to seven for SM, GNS and BIO. For 38 QTLs, additive effect was positive and for 47 QTLs was negative, indicating inheritance to the offspring of favorable alleles in theses loci from either #49 or Yecora Rojo, respectively. Five QTLs were stably identified across irrigated and water deficit conditions. The chromosomes 4A and 6B possessed QTLs associated with most of the traits (PH, SL, GNS, BIO and GY) and (PH, SM, TKW and GY), respectively. Genetic positions of yield-related trait QTLs are shown in Figure 3a–c.

#### 2.4.1. Grain Yield (GY)

In total, eight QTLs were detected for grain yield with LOD ranging from 3.49 to 4.22 and R^2^ values from 8.30 to 10.5% (Table 3, Figure 3a–c). Among these, six QTLs on chromosomes 2A, 4A and 6B (4) and two QTLs on chromosome 2A were identified under irrigated and water deficit conditions, respectively. The favorable alleles for GY at the loci on chromosomes 4A and 6B were contributed by #49, and those on chromosome 2A were inherited from Yecora Rojo.

#### 2.4.2. Plant Height (PH)

For plant height, 10 QTLs on chromosomes 2B, 3A, 3D, 4B (2), 4D, 6B (2), 7A and 7B with LOD ranging from 3.59 to 10.60 and R^2^ from 6.20 to 19% were mapped under irrigated conditions (Table 3, Figure 3a–c). Under water deficit conditions, nine QTLs for PH were identified on chromosomes 4A, 4B, 4D, 7A (5) and 7D with LOD ranging from 3.81 to 6.13 explaining 8.9–14.0% of the trait phenotypic variance. The favorable alleles were contributed by Yecora Rojo at all loci except for QTL on chromosome 6B under water deficit conditions. The two QTLs on chromosome 4B and 4D were consistent under both conditions.

#### 2.4.3. Number of Spikes per Square Meter (SM)

Six QTLs on chromosomes 5A, 2B and 6B under irrigated and one QTL on chromosomes 2B under water deficit conditions were mapped. The LOD scores ranged from 3.68 to 8.01 with R^2^ value ranging from 8.70 to 17.6%. The #49 contributed the favorable alleles at all the loci except for QTLs on chromosome 2B (Table 3, Figure 3a–c).

#### 2.4.4. 1000. Kernel Weight (TKW)

For 1000 kernel weight, 13 QTLs with LOD ranging from 3.32 to 5.17 were identified (Table 3, Figure 3a–c). QTLs identified under irrigated conditions were mapped to chromosomes 1B (3), 5B (2) and 6B explaining 7.90–14.61% of phenotypic variance and seven QTLs identified under water deficit conditions were allocated to chromosomes 3B (2), 4B (2), 5B (2) and 7B explaining 8.30 to 11.60% phenotypic variation. For TKW, favorable alleles at the identified QTLs were contributed by both parents.

#### 2.4.5. Number of Grains per Spike (NGS)

Seven QTLs were identified for number of grains per spike on chromosomes 1A, 3A, 4A (3) and 7D (2) with LOD score from 2.82 to 7.58 and R^2^ from 6.6 to 19.25% and both parents contributed the favorable alleles (Table 3, Figure 3a–c). Among these, three QTLs were found under irrigated and four QTLs under water deficit conditions. The QTL on 4A was stable at two locations and under two conditions.

#### 2.4.6. Spike Length (SL)

Spike length was affected by nine QTLs mapped to chromosomes 2A, 2B, 3A, 3B, 4A (2), 7A and 7D (2) with R^2^ from 7.40 to 19.18% and LOD score from 3.27 to 7.15 under two conditions (Table 3, Figure 3a–c). Both parents contributed favorable alleles at the identified QTLs.

#### 2.4.7. Biomass (BIO)

Totally seven QTLs on 1B (2), 1D, 4A (2) and 5A (2) were associated significantly with biomass (Table 3, Figure 3a–c). BIO QTLs explained the phenotypic variation with an average 10.16% and their LOD score varied from 3.65 to 4.43. Except for loci on 5A, #49 contributed alleles for increasing biomass.

#### 2.4.8. Harvest Index (HI)

For the harvest index, we detected seven QTLs on 1B (2), 2A (2), 3D and 6A (2) and eight QTLs on 1B (2), 2D, 3B (4) and 5A under irrigated and water deficit conditions, respectively (Table 3, Figure 3a–c). HI QTLs explained an average of 10.63% phenotypic variation and the LOD score ranged from 2.55 to 6.43. Both parents contributed alleles to affect this trait.

## 3. Discussion

Improving grain yield under water deficit conditions is of utmost importance for wheat breeding programs for arid and semi-arid regions of the world. To study drought tolerance related genomic regions, QTL mapping has been conducted using GBS in a RIL population derived from a cross between #49 × Yecora Rojo under irrigated and water deficit conditions. Due to simultaneous discovery and genotyping of a large numbers of SNPs, cost-efficiency and flexibility, GBS has been applied to mapping of QTLs in several plant species including wheat [21,32,33,34,37,38]. We employed GBS to genotype 148 recombinant inbred lines and two parental lines. After filtering against low-quality markers, 7788 high quality SNPs were used to generate a linkage map.

In the present study, 85 putative QTLs for grain yield and its related traits were identified on 19 out of 21 chromosomes of hexaploid wheat (Table 3, Figure 3a–c). We could identify unique QTLs for studied traits that were likely not detected in the previous studies. This population revealed high-effect QTL under water deficit conditions for spike length in the interval of 59.9–75.5 Mb of 7A with 19.18% R^2^ and 7.15 LOD score (*Qsl-Wd.mah-7A*) and favorable alleles were contributed by Yecora Rojo. SL QTL *Qsl-Wd.mia-2B* detected on 2B spanning from 26.5 to 33.7 Mb (with 11.64% R^2^) has not also been reported. For TKW under water deficit conditions, two unique QTLs, *Qtkw-Wd.mah-3B* and *Qtkw-Wd.mia-3B,* with 11.59 and 8.60% R^2^, were identified on 3B (20.4–28 Mb). GNS QTL, *Qgns-Wd.mia-1A*, was mapped on 1A (12–14.8 Mb) and explained the 11.10% phenotypic variation. The genomic regions on chromosome 1B contained BIO QTLs (*Qbio1-Wd.mia-1B* and *Qbio2-Wd.mia-1B*) spanning from 9.6 to 16.4 Mb and HI QTLs (*Qhi1-Wd.tab-1B* and *Qhi2-Wd.tab-1B*) spanning from 420.5 to 427 Mb. In this study, some unique QTLs related to irrigation conditions were also identified, the high effect of which was *Qhi-Irr.mia-6A* (580–595 Mb) with a 14.20% R^2^ and 6.43 LOD score. Two QTLs on chromosome 6B spanning from 28 to 35 Mb (43 and 49 cM) were co-localized for GY and SM under irrigation conditions that explained an average of 9.28% phenotypic variation, and the LOD score ranged from 3.65 to 4.22. BIO QTL (*Qbio-Irr.mia-1D*) on chromosome 1D (34–250 Mb) and PH QTL (*Qph-Irr.3P-3A*) on chromosome 3A (33.4–37.2 Mb) were identified under irrigation conditions and explained 10.78 and 8.30% of the phenotypic variation, respectively. To date, numerous QTL associated with grain yield and yield components have been reported by using various populations. However, different QTL expression and its position may be influenced by factors such as type and size of population, density of the linkage map, analytical tools and the environmental conditions used in different studies [39].

The corresponding loci that were previously reported for yield-related traits in the same interval were extracted from literature and are shown in Supplementary Table S2 and Figure S1. This study mapped TKW QTLs (*Qtkw1-Irr.mah-1B*, *Qtkw2-Irr.mah-1B* and *Qtkw-Irr.mia-1B*) in the interval of 639–646.1 Mb of chromosome 1B explained the phenotypic variation with an average 12.74%, which were reported for QTLs associated with TKW and GY [6,40]. In the interval of 20–33 Mb of 2A, three GY (*Qgy-Irr.mia-2A*, *Qgy-Wd.mia-2A* and *Qgy-Wd.3P-2A*) and a SL (*Qsl-Wd.3P-2A*) QTLs were found, in which QTLs associated with GY and PH have been reported in the literature [34,41,42]. We mapped SM QTL (*Qsm-Wd.tab-2B*) on 2B spanning from 493.6 to 548.6 Mb, which have been reported for PH and GY [10,43]. Identified QTLs for PH (*Qph-Irr.3P-2B*) and SM (*Qsm1-Irr.3P-2B* and *Qsm2-Irr.3P-2B*) on chromosome 2B (770–780.5 Mb) were also in a similar region of the PH QTLs reported by Zanke et al. [42]. *Qgns-Irr.mah-3A* and *Qsl-Irr.tab-3A* were mapped on 695.6–707.9 Mb of chromosome 3A, and this loci could correspond to the QTL for GY identified by Bhatta et al. [6]. A region of 3B (37.6–44.8 Mb) contained SL QTL *Qsl-Wd.mah-3B* explained 8.8% variation in SL corroborate with the findings of Li at al. [17]. We identified a 6 Mb region on 3D (453.9–459.2 Mb) associated with PH and HI, of which the HI QTL was also mapped in a similar region by Bhatta et al. [6]. In addition to the grain yield QTLs, a set of QTLs associated with PH and BIO was identified on chromosomes 4A. QTL cluster on chromosome 4A includes *Qgy-Irr.mah-4A*, *Qph-Wd.3P-4A*, *Qbio-Irr.mah-4A* and *Qbio-Irr.3P-4A* were mapped in a flanking region from 612 to 621 Mb, which explained the phenotypic variation with an average 9.5%. Previously, QTLs for grain yield and its components have been localized in this interval [6,9,13,14,16,18,44]. A region on 4A (632.2–684.9 Mb) contained QTLs for GNS (*Qgns1-Irr.tab-4A*, *Qgns2-Irr.tab-4A* and *Qgns-Wd.3P-4A*) and for SL (*Qsl1-Irr.tab-4A* and *Qsl2-Irr.tab-4A*) with an average of 14.32% and 8.92% phenotypic variation, respectively. QTLs at similar locations were previously reported for PH [10], GNS and TKW [15]. Several significant QTLs for PH (*Qph1-Irr.tab-4B*, *Qph2-Irr.tab-4B* and *Qph-Wd.tab-4B*) and for TKW (*Qtkw1-Wd.tab-4B* and *Qtkw2-Wd.tab-4B*) were detected in the interval of 21.5–37.5 Mb of 4B, explaining 8.4–19% of phenotypic variation. This region contains *Rht-B1* gene regulating plant height. Previously, QTLs associated with PH, SL, GY and GNS have been reported in this region [15,17,24,29,33]. In the interval of 112–342.7 Mb on 4D, PH QTLs (*Qph-Irr.tab-4D* and *Qph-Wd.tab-4D*) were mapped with R^2^ value ranging from 9.04 to 18.6%. This genomic region, containing *Rht-D1* gene, have been reported by several studies for carrying QTLs associated with PH, GY, BIO and TKW [23,28,45,46,47]. These two genomic regions on 4B and 4D were detected under both irrigated and water deficit conditions with strong effects on plant height, and alleles contributed by Yecora Rojo reduced plant height. Basically, dwarfing is one of the morphological responses to drought stress [48]. Besides the *Rht* loci, genes required for vernalization response (*Vrn*) have also attracted attention of breeding to improve plant adaptation by flowering at the appropriate time. Therefore, identifying QTLs for yield-related traits close to *Vrn* genes can be predicted [11]. In the present study, QTLs associated with SM *Qsm-Irr.tab-5A* and *Qsm-Irr.3P-5A* explaining 13.8 to 17.6% variation, mapped closely to the *VrnA1* gene on chromosome 5A (581–589 Mb). Likewise, some QTLs associated with various traits have been previously reported in this genomic region [42,49]. Genomic regions spanning from 360.4–445 Mb on chromosome 5B were detected controlling TKW. The identified QTLs (*Qtkw1-Irr.tab-5B*, *Qtkw2-Irr.tab-5B*, *Qtkw1-Wd.tab-5B* and *Qtkw2-Wd.tab-5B*) explained 8.30 to 13% of phenotypic variation. QTLs associated with TKW and GY have been localized in the similar interval of 5B by previous studies [15,46,50]. The present study also confirmed QTLs for TKW (*Qtkw-Irr.tab-6B*) and PH (*Qph1-Irr.mia-6B* and *Qph2-Irr.mia-6B*) in 659.5–676 Mb of 6B, which have been previously reported [8,28]. Under water deficit conditions, five PH QTLs (*Qph1-Wd.mia-7A*, *Qph2-Wd.mia-7A*, *Qph1-Wd.3P-7A*, *Qph2-Wd.3P-7A* and *Qph3-Wd.3P-7A*) were mapped in the interval of 15.5–28.5 Mb of 7A explaining 8.9–11.4% variation in PH. Acuña-Galindo et al. [51] using SNP markers identified BIO QTL in the similar interval under drought and heat stress. A QTL associated with PH (*Qph-Irr.3P-7B*) was mapped on 7B (648.7–657.8 Mb) which reported by Zanke et al. [42] for plant height. A region on 7B spanning from 693–703.9 Mb have been previously associated with TKW [40], PH [52] and GNS [14], which co-localized with TKW QTL in this study under water deficit stress (*Qtkw-Wd.tab-7B*). QTLs associated with GNS (*Qgns1-Wd.mah-7D*) and SL (*Qsl1. Irr.tab-7D* and *Qsl2. Irr.tab-7D*) were identified in the interval of 89.3–105 Mb of 7D explained 6.6–9.10% of phenotypic variation. This locus was already detected controlling GY [28], GNS [14,17], PH [42] and TKW [40,53].

We aimed to predict candidate genes for QTL function on the basis of the structural and functional gene annotation provided with Chinese Spring RefSeqv1.0 [36]. The important putatively drought-related genes underlying the QTL intervals are listed in Table 4. Since the physical intervals of several QTLs overlapped with each other, the same annotated gene is co-located with more than one QTL.

Potential candidate genes underlying *Qtkw-Wd.tab-7B*, *Qsl1-Irr.tab-4A* and *Qph1-Irr.mia-6B* were annotated as F-box family protein. F-box proteins play a role in responses to hormones [54,55], light and abiotic stress [56,57]. Other putatively drought-related genes for PH QTLs on chromosomes 4B under irrigated and water deficit conditions belonged to the gene class of protein kinases. Generally, protein kinases are involved in drought response as regulatory proteins. Mao et al. [58] reported that a serine/threonine-protein kinase of wheat increases multi-stress tolerance in Arabidopsis. The gene underlying *Qph1-Wd.mia-7A* and *Qph1-Wd.3P-7A* identified under water deficit condition was a 60 kDa chaperonin protein. The 60 kDa chaperonin, like other heat-shock proteins, are induced by hot temperatures, salinity, cold and water deficit stress as a protective mechanism [59,60]. NBS-LRR disease resistance protein genes were present in chromosomal regions of two QTLs under water deficit condition for grain yield and spike length on chromosomes 2A and 2B, respectively. Improvement of drought tolerance by enhanced expression of NBS-LRR genes was reported by Chini et al. [61]. The function of candidate genes underlying *Qtkw-Wd.mah-3B* and *Qsl-Wd.3P-2A* were annotated as AP2-EREBP transcription factor and ABC transporter protein, respectively. Liu and et al. [62] and Song and et al. [63] reported the role of an AP2-EREBP transcription factor in signal transduction pathways in drought and ABA responses. Furthermore, some members of the ABC transporter family proteins mediate the transport of acyl-coenzyme A [64] which contributes to drought tolerance. An NAD(P)H-quinone oxidoreductase was located within marker intervals for GNS QTLs (*Qgns2-Irr.tab-4A*, *Qgns-Wd.3P-4A* and *Qgns2-Irr.tab-4A*) on chromosome 4A. NAD(P)H-quinone oxidoreductase inhibits the production of semiquinones and oxygen radicals by the reduction of quinones to quinols [65]. Cysteine proteinase genes were co-located with grain yield QTLs on chromosome 6B identified in irrigated condition. The role of cysteine proteinases family in wheat under severe drought [66] and salinity, drought, oxidation and cold stress [67] has been established. Another important response to abiotic stress particularly drought is the elimination of reactive oxygen species (ROS) which is performed by different kind of detoxification proteins. In present study, a gene related to detoxification function was found within QTL intervals for PH on chromosome 7A (*Qph-Irr.tab-7A*). Cytochrome P450 genes were identified for HI QTLs intervals on chromosomes 2A (*Qhi2-Irr.mia-2A*) and 1B (*Qhi2-Irr.tab-1B*). Cytochrome P450 genes are members of a large superfamily enzyme which involved in drought stress response [68]. Ubiquitin-conjugating enzyme E2 was annotated on QTLs underlying 1000 kernel weight on chromosome 1B (*Qtkw1-Irr.mah-1B* and *Qtkw-Irr.mia-1B*). Ubiquitinylation is a multi-step reaction for protein degradation and plays an important role in light signaling, biotic and abiotic stress responses [69].

## 4. Materials and Methods

### 4.1. Plant Materials

The mapping population consisted of 148 F8 recombinant inbred lines (RILs) derived from a cross between genotype Iran #49 and Yecora Rojo. Iran #49 is a tall late spring landrace collected at Allary, 30°56′, 61°39′, alt. 530 m, average rainfall = 50 mm, in Bluchestan, southeast Iran with a large root system. Yecora Rojo is spring wheat variety from Mexico which was cultivated in Southern California for more than 40 years, which is carrying two dwarfing genes and is characteristic for its shallow root system [35]. The parental lines were different for a number of phenological, morphological, and agronomic traits including grain yield and plant height.

### 4.2. Experimental Design and Phenotypic Evaluation

Field trials were conducted during growing seasons of the years 2008–2009 and 2009–2010 at the University of Tabriz, Faculty of Agriculture research farm (N 46°, 17′ E 38°, 05′ and 1360 m), and in the growing seasons of the years 2013–2014 and 2014–2015 at the PayamNour University of Mahabad, Mahabad research station (N 45°43′, E 36°01′ and 1320 m) and Miandoab Agricultural and Natural Resource Station farm (N 46°06′, E 36°58′ and 1314 m), respectively, under irrigated and water deficit conditions at the flowering stage, resulting in a total of 12 environments. In each station, the 148 RILs and two parental lines were planted in an alpha lattice design with two replications each consisting of ten incomplete blocks with 15 genotypes, both under water deficit and irrigation conditions. Each line was sown in three rows of 2.5 m length with inter- and in-row spacing of 20 and 5 cm, respectively. Irrigation in non-stress conditions was done after 70 mm evaporation from class A Pan corresponded to soil water potential of −0.5 MPa and in water deficit conditions was performed after 130 mm evaporation from class-A Pan corresponded to soil water potential of −1.2 MPa. In the stressed treatment, water deficit stress was induced by stopping irrigation from 50% heading to physiological maturity in order to simulate terminal drought stress. The following phenotypic traits were recorded: The number of spikes per square meter (SM) was recorded at physiological maturity and plant height (PH) was measured in centimeters (cm) from the ground to the tip of the spike from 10 randomly sampled and tagged plants in each plot before harvesting. The spike length (SL) [measured in cm] and the numbers of grains per spike (NGS) were recorded after harvesting from the main tillers of 10 randomly selected plants. Thousand kernel weight (TKW) was determined measured from randomly sampled 1000 seeds after harvest and expressed in g/1000 seed. The grain yield per plot (GY) and biomass were determined as the weight (grams) of the grain from a plot where the plot sizes were 2.5 m rows with 50 plants after eliminating borders. Finally, the harvest index (HI) was calculated as the proportion of the total biomass devoted to grain yield.

### 4.3. GBS Library Construction, Genotyping and SNP Calling

DNA was extracted and purified from the leaf tissue of the RILs and parental lines using Guanidine thiocyanate-based DNA isolation technique. Briefly, six centimeters of two-week-old of leaves were harvested and transferred into 1.1 mL 8-strip mini tubes (supplier, type) together with two 4mm glass beads and homogenized by using a 96-well block holder and a Retsch MM 400 mixer mill (Retsch GmbH, Haan, Germany) for a minimum of 1 min at 30 Hz frequency. 600 μL preheated GTC extraction buffer (1 M Guanidine thiocyanate, 2 M NaCl, 30 mM NaAc pH 6.0) was added to the tubes and incubated at 65 °C for 30 min. After spinning for 30 min at 4000 rpm using a table top centrifuge, 480 μL of the supernatants were transferred to a 96-Well AcroPrep Advance plate (Thermo Fisher Scientific GmbH, Dreieich, Germany) for vacuum filtration. 900 μL wash buffer (50 mM NaCl, 10 mM Tris/HCl pH 8, 1mM EDTA, Ethanol 70%) was added twice and the vacuum was applied with a vacuum manifold after every washing step. The 96-Well AcroPrep Advance plate was placed onto a NUNC 96-well plate (Thermo Fisher Scientific GmbH, Dreieich, Germany) and 100 μL preheated 1x TE light elution buffer (0.1 mM EDTA, 10 mM Tris/HCl pH 8) was pipetted to each well. Extracted DNA was eluted by spinning at 3500 rpm for 10 min. DNA quality and concentration were checked by running electrophoresis of 1% agarose gels (Invitrogen, Carlsbad, CA, USA) in 1X TBE buffer (89 mM Tris, 89 mM Boric acid, 2 mM EDTA pH 8.0) and by using Qubit 2.0 Fluorometer (Invitrogen, Carlsbad, CA, USA) were used.

GBS libraries were constructed for each RIL and parental lines according to a previously described protocol [70]. Libraries comprising 150 individually barcoded samples were sequenced as a pool in three lanes of an Illumina HiSeq2500 (Illumina Inc., San Diego, CA, USA). The length of single-end reads was 100 bp. Each of the RILs and parents were sequenced three and six times, respectively, to specify the minimum required number of reads per sample. Raw data processing followed a previously established reference-based GBS pipeline using SAMtools [71]. First, sequence reads were quality- and adaptor-trimmed and then mapped against Chinese Spring RefseqV1.0 [36]. Variant filtering was done with the following parameters: minimum minor allele frequency 0.05, maximum fraction of missing genotype call 0.9 and minimum read depth 4 for both of homozygous and heterozygous genotype calls. As the maximum expected residual heterozygosity for RIL population is 10%, this parameter was set to 0.1. Identified SNPs were named as “chromosome name_ physical position” i.e., chr4A_614700608. All unknown or heterozygous SNPs in the parents as well as those with >20% missing data were excluded from further analysis.

### 4.4. Linkage Map Construction and QTL Mapping

A total of 7788 SNPs were used for construction of a linkage map based on 148 RILs. Linkage analysis was performed using the regression mapping function in JoinMap 4.1 (Kyazma B.V., Wageningen, The Netherlands) [72] with LOD values in the range from 3 to 8. The Kosambi mapping function was used to convert recombination frequency to genetic distance based on centi-Morgan [73]. The identical markers and unmapped SNPs were removed automatically by JoinMap. The loci with significant segregation distortion (*p* ≤ 0.01) were also excluded. QTLs were identified by composite interval mapping (CIM) based on model 6 and forward and backward regression to select cofactors implemented in WinQTL Cartographer V2.5 software [74]. The QTL threshold for the studied traits (LOD significance threshold) was estimated by 1000 permutations. The percentage of phenotypic variance explained by each QTL and their additive effect were also evaluated. Each QTL was denominated as “Q” (abbreviation of QTL)+ trait name+ trial name+ chromosome name. For example, *Qtkw-Wd.mia-3B* reveals QTL for thousand kernel weight under water deficit conditions in Miandoab in which it stands on 3B chromosome. MapChart (version 2.3) software [75] was used for the graphical presentation of linkage maps and QTL positions. The physical position of flanking markers surrounding identified QTLs were extracted and used to search on the IWGSC_RefSeq v1.0 assembly. Gene IDs present in each of physical intervals were obtained from JBrowse. The annotation of the gene was then retrieved from the Functional Annotation_v1 of IWGSC_RefSeqv1.0, which is publicly available at https://wheat-urgi.versailles.inra.fr/Seq-Repository/Annotations (accessed on 21 October 2018).

## 5. Conclusions

The results of present study indicate that GBS methods provide an opportunity to develop a high-density linkage map for the RIL population derived from a cross between #49 and Yecora Rojo, and precise identification of QTLs for grain yield and its components. For grain yield and its related traits 20 novel QTLs explaining 8.87–19.18% of phenotypic variance were mapped on chromosomes 1A, 1B, 1D, 2B, 3A, 3B, 6A, 6B and 7A. We could identify two stable QTLs for plant height under irrigated and water deficit conditions, which they were associated with *Rht-B1* and *Rht-D1* genes on chromosomes 4B and 4D, respectively. Some linkage groups had QTLs associated with several traits showing pleiotropic effects, which might be interesting for gene pyramiding. In addition, annotation of genes underlying the QTL intervals revealed their potential role in drought stress responses. These QTLs and potential candidate genes can be further analyzed and validated for breeding applications.

## Figures and Tables

**Table 1:** Pearson’s correlation coefficients between studied traits under irrigated and water deficit conditions.

	Water Condition	PH	SL	SM	GNS	TKW	BIO	GY	HI
PH	irrigated	1							
	water deficit	1							
SL	irrigated	0.269 **	1						
	water deficit	0.358 **	1						
SM	irrigated	−0.197 *	−0.238 **	1					
	water deficit	−0.088	−0.141	1					
GNS	irrigated	0.321 **	0.517 **	−0.174 *	1				
	water deficit	0.242 **	0.486 **	−0.103	1				
TKW	irrigated	0.165 *	0.141	0.057	0.170 *	1			
	water deficit	0.112	0.027	−0.038	0.073	1			
BIO	irrigated	0.439 **	0.354 **	0.253 **	0.336 **	0.346 **	1		
	water deficit	0.344 **	0.290 **	0.351 **	0.322 **	0.244 **	1		
GY	irrigated	0.276 **	0.261 **	0.330 **	0.451 **	0.423 **	0.640 **	1	
	water deficit	0.149	0.207 *	0.295 **	0.385 **	0.421 **	0.561 **	1	
HI	irrigated	0.008	0.1	0.198 *	0.314 **	0.281 **	0.019	0.755 **	1
	water deficit	−0.086	0.058	0.082	0.217 **	0.292 **	−0.102	0.742 **	1

Correlation coefficients between the averaged traits across three locations and two years are shown on top for irrigated condition and on bottom for water deficit condition. * and ** significant at a = 0.05 and a = 0.01 level (2-tailed).

**Table 2:** Summary of the GBS markers mapped to the 21 chromosomes based on 148 recombinant inbred lines derived from a cross between #49 and Yecora Rojo.

Chromosome	No. SNP	No. SNP after Filtering	Mapped Loci to Linkage Group	Length of Linkage Group (cM)	Distance between Two Adjacent Markers	SNP per cM
1A	2080	476	399	189.22	0.47	2.11
2A	1868	451	356	231.32	0.65	1.54
3A	1447	342	267	208.69	0.78	1.28
4A	2135	396	335	220.39	0.66	1.52
5A	1767	424	343	276.68	0.81	1.24
6A	1305	323	254	179.48	0.71	1.42
7A	2730	630	500	260.88	0.52	1.92
A genome	13,332	3042	2454	1566.64		
1B	2520	548	419	190.72	0.46	2.20
2B	3384	785	570	268.49	0.47	2.12
3B	3516	707	540	257.19	0.48	2.10
4B	1164	222	180	131.50	0.73	1.37
5B	2499	492	380	281.99	0.74	1.35
6B	2926	636	422	162.63	0.39	2.59
7B	2828	587	422	227.19	0.54	1.86
B genome	18,837	3977	2933	1519.70		
1D	601	159	101	115.38	1.14	0.88
2D	837	237	126	152.11	1.21	0.83
3D	268	43	29	83.70	2.89	0.35
4D	134	27	15	12.89	0.86	1.16
5D	276	49	29	18.17	0.63	1.60
6D	498	128	49	9.19	0.19	5.33
7D	622	126	95	164.36	1.73	0.58
D genome	3236	769	444	555.80		
Total	35,405	7788	5831	3642.14	0.62	1.60

**Table 3:** List of significant QTL detected for grain yield (GY), plant height (PH), spike number per squared meters (SM), thousand kernel weight (TKW), grain number per spike (GNS), spike length (SL), biomass (BIO), and harvest index (HI) in the Iran #49 × Yecora Rojo RIL population under irrigated and water deficit conditions.

Trait	Water Condition	Environment	QTL	Closest Marker	Position (cM)	LOD	R^2^ (%) ^a^	Add ^b^
GY	Irrigated	Mahabad	*Qgy-Irr.mah-4A*	chr4A_614700608	82	3.92	8.57	12.94
			*Qgy1-Irr.mah-6B*	chr6B_33629751	43	3.80	8.87	11.76
			*Qgy2-Irr.mah-6B*	chr6B_34082536	49	3.65	8.50	11.51
		Miandoab	*Qgy-Irr.mia-2A*	chr2A_30568266	43	3.98	9.21	−12.01
		Mean of 3 Places	*Qgy1-Irr.3P-6B*	chr6B_33629751	43	4.22	9.90	9.63
			*Qgy2-Irr.3P-6B*	chr6B_34340678	49	4.06	9.50	9.39
	Water deficit	Miandoab	*Qgy-Wd.mia-2A*	chr2A_24214196	34	4.21	10.50	−9.10
		Mean of 3 Places	*Qgy-Wd.3P-2A*	chr2A_24214196	34	3.49	8.30	−5.97
PH	Irrigated	Mahabad	*Qph-Irr.mah-3D*	chr3D_453986741	24	3.59	9.78	2.42
		Miandoab	*Qph1-Irr.mia-6B*	chr6B_567765886	109	3.64	8.6	−3.62
			*Qph2-Irr.mia-6B*	chr6B_659589376	129	4.61	11.42	4.43
		Tabriz	*Qph1-Irr.tab-4B*	chr4B_28735878	44	7.21	14.00	−2.96
			*Qph2-Irr.tab-4B*	chr4B_37347202	50	10.60	19.00	−3.45
			*Qph-Irr.tab-4D*	chr4D_120094414	5	10.22	18.60	−3.40
			*Qph-Irr.tab-7A*	chr7A_83776793	113	3.79	6.20	−1.97
		Mean of 3 Places	*Qph-Irr.3P-2B*	chr2B_770220840	239	3.82	9.30	1.61
			*Qph-Irr.3P-3A*	chr3A_35421232	62	3.60	8.30	−1.51
			*Qph-Irr.3P-7B*	chr7B_650619590	140	3.65	8.30	1.54
	Water deficit	Mahabad	*Qph-Wd.mah-7D*	chr7D_485517060	110	4.24	14.00	−2.65
		Miandoab	*Qph1-Wd.mia-7A*	chr7A_16302867	16	3.88	9.10	−1.86
			*Qph2-Wd.mia-7A*	chr7A_22919790	26	3.81	8.90	−1.83
		Tabriz	*Qph-Wd.tab-4B*	chr4B_28735878	44	6.13	12.70	−2.60
			*Qph-Wd.tab-4D*	chr4D_120094414	2	4.67	9.04	−2.20
		Mean of 3 Places	*Qph-Wd.3P-4A*	chr4A_614700608	76	4.06	9.30	1.48
			*Qph1-Wd.3P-7A*	chr7A_16302867	16	4.27	10.00	−1.42
			*Qph2-Wd.3P-7A*	chr7A_18775675	22	4.90	11.40	−1.50
			*Qph3-Wd.3P-7A*	chr7A_22919790	27	4.24	10.50	−1.40
SM	Irrigated	Mahabad	*Qsm1-Irr.mah-6B*	chr6B_33629751	43	3.81	9.46	9.49
			*Qsm2-Irr.mah-6B*	chr6B_34340678	49	3.81	9.46	9.47
		Tabriz	*Qsm-Irr.tab-5A*	chr5A_589287461	166	8.01	17.60	24.26
		Mean of 3 Places	*Qsm1-Irr.3P-2B*	chr2B_777297032	247	3.68	8.80	−8.68
			*Qsm2-Irr.3P-2B*	chr2B_780590240	252	4.02	9.50	−8.50
			*Qsm-Irr.3P-5A*	chr5A_581488651	165	5.81	13.80	10.45
	Water deficit	Tabriz	*Qsm-Wd.tab-2B*	chr2B_513284737	128	3.97	8.70	−14.64
TKW	Irrigated	Mahabad	*Qtkw1-Irr.mah-1B*	chr1B_640559774	145	5.17	14.61	−3.91
			*Qtkw2-Irr.mah-1B*	chr1B_642697116	150	4.78	12.04	−3.42
		Miandoab	*Qtkw-Irr.mia-1B*	chr1B_642697116	150	4.62	11.57	−3.67
		Tabriz	*Qtkw1-Irr.tab-5B*	chr5B_339425357	64	5.73	13.00	1.29
			*Qtkw2-Irr.tab-5B*	chr5B_378853109	74	4.44	10.00	1.09
			*Qtkw-Irr.tab-6B*	chr6B_668966788	139	3.40	7.90	0.93
	Water deficit	Mahabad	*Qtkw-Wd.mah-3B*	chr3B_21770957	44	4.18	11.59	2.51
		Miandoab	*Qtkw-Wd.mia-3B*	chr3B_20721650	42	3.39	8.60	1.79
		Tabriz	*Qtkw1-Wd.tab-4B*	chr4B_21573529	37	4.35	11.60	−1.08
			*Qtkw2-Wd.tab-4B*	chr4B_37347202	50	3.79	8.40	−0.90
			*Qtkw1-Wd.tab-5B*	chr5B_412238630	77	4.48	11.00	1.08
			*Qtkw2-Wd.tab-5B*	chr5B_430752407	85	3.32	8.30	0.92
			*Qtkw-Wd.tab-7B*	chr7B_701649237	173	3.90	8.80	−0.91
GNS	Irrigated	Mahabad	*Qgns-Irr.mah-3A*	chr3A_695662512	150	4.19	8.13	−0.65
		Tabriz	*Qgns1-Irr.tab-4A*	chr4A_632236000	122	7.58	19.25	1.61
			*Qgns2-Irr.tab-4A*	chr4A_683874492	129	6.27	14.60	1.40
	Water deficit	Mahabad	*Qgns1-Wd.mah-7D*	chr7D_94715776	85	2.82	6.60	0.65
			*Qgns2-Wd.mah-7D*	chr7D_180083703	98	4.04	10.00	−0.77
		Miandoab	*Qgns-Wd.mia-1A*	chr1A_11934211	22	4.29	11.10	−0.68
		Mean of 3 Places	*Qgns-Wd.3P-4A*	chr4A_683874492	130	3.64	9.10	0.51
SL	Irrigated	Tabriz	*Qsl-Irr.tab-3A*	chr3A_697615272	158	4.74	11.92	−0.26
			*Qsl1-Irr.tab-4A*	chr4A_639994434	124	3.45	7.40	0.21
			*Qsl2-Irr.tab-4A*	chr4A_681683160	132	4.61	10.43	0.25
			*Qsl1-Irr.tab-7D*	chr7D_89732435	80	4.01	9.10	−0.23
			*Qsl2-Irr.tab-7D*	chr7D_104889647	89	4.61	9.70	−0.24
	Water deficit	Mahabad	*Qsl-Wd.mah-3B*	chr3B_43100457	76	3.80	8.80	−0.21
			*Qsl-Wd.mah-7A*	chr7A_66641918	94	7.15	19.18	−0.25
		Miandoab	*Qsl-Wd.mia-2B*	chr2B_26987136	46	3.97	11.64	0.17
		Mean of 3 Places	*Qsl-Wd.3P-2A*	chr2A_24111229	25	3.27	8.20	−0.11
BIO	Irrigated	Mahabad	*Qbio-Irr.mah-4A*	chr4A_614700608	77	3.77	9.42	17.03
		Miandoab	*Qbio-Irr.mia-1D*	chr1D_34011022	55	3.65	10.78	18.86
		Mean of 3 Places	*Qbio-Irr.3P-4A*	chr4A_614700608	78	3.98	10.70	15.66
	Water deficit	Mahabad	*Qbio-Wd.mah-5A*	chr5A_552523257	128	3.84	10.38	−16.00
		Miandoab	*Qbio1-Wd.mia-1B*	chr1B_9691095	9	3.67	9.30	13.95
			*Qbio2-Wd.mia-1B*	chr1B_15368052	17	3.84	9.60	14.41
		Mean of 3 Places	*Qbio-Wd.3P-5A*	chr5A_552523257	131	4.43	10.95	−11.43
HI	Irrigated	Mahabad	*Qhi-Irr.mah-3D*	chr3D_197992771	23	3.72	9.70	2.14
		Miandoab	*Qhi1-Irr.mia-2A*	chr2A_3975444	5	3.67	8.10	−2.20
			*Qhi2-Irr.mia-2A*	chr2A_6269139	14	5.02	11.77	−2.61
			*Qhi-Irr.mia-6A*	chr6A_591883833	122	6.43	14.20	−2.91
		Tabriz	*Qhi1-Irr.tab-1B*	chr1B_420591625	77	4.87	11.60	−2.71
			*Qhi2-Irr.tab-1B*	chr1B_498063961	87	5.09	12.10	−2.64
		Mean of 3 Places	*Qhi-Irr.3P-6A*	chr6A_591883833	122	4.42	9.70	−1.47
	Water deficit	Mahabad	*Qhi-Wd.mah-2D*	chr2D_618150012	142	2.55	6.06	1.96
			*Qhi1-Wd.mah-3B*	chr3B_178266388	109	2.86	7.25	2.32
			*Qhi2-Wd.mah-3B*	chr3B_419560745	117	4.57	11.54	2.98
			*Qhi3-Wd.mah-3B*	chr3B_492105670	125	5.06	12.47	3.19
			*Qhi4-Wd.mah-3B*	chr3B_728922412	159	3.57	8.90	−2.54
		Miandoab	*Qhi-Wd.mia-5A*	chr5A_52357876	63	5.33	13.00	−3.85
		Tabriz	*Qhi1-Wd.tab-1B*	chr1B_420591625	77	4.89	11.50	−2.74
			*Qhi2-Wd.tab-1B*	chr1B_427069393	81	4.89	11.50	−2.74

^a^: Phenotypic variance explained (%) for each QTL; ^b^: Negative and positive additive effects for detected QTLs are indicating the Yecora Rojo and #49 contributes alleles to increase the traits respectively.

**Table 4:** List of putatively drought-related genes underlying QTL detected in the RIL population of #49 × Yecora Rojo population under irrigated and water deficit conditions.

QTL Name	Flanking Markers ^a^	Gene-ID	Annotation
*Qtkw1-Irr.mah-1B*	chr1B_637810003-chr1B_641627897	TraesCS1B01G415500.1	Ubiquitin-conjugating enzyme E2
*Qtkw-Irr.mia-1B*	chr1B_639448207-chr1B_642697116		
*Qtkw-Wd.mia-3B*	chr3B_20439595-chr3B_22054094	TraesCS3B01G041700.1	Alpha-glucosidase
*Qtkw-Wd.mah-3B*	chr3B_20439595-chr3B_22054094	TraesCS3B01G042400.1	AP2-EREBP transcription factor
*Qtkw-Wd.tab-7B*	chr7B_692926289-chr7B_702176728	TraesCS7B01G434600.1	FBD-associated F-box protein
*Qgy-Wd.mia-2A*	chr2A_20237446-chr2A_33006222	TraesCS2A01G057700.1	NBS-LRR disease resistance protein
*Qgy-Wd.3P-2A*			
*Qgy-Irr.mia-2A*			
*Qgy2-Irr.3P-6B*	chr6B_28012560-chr6B_35789585	TraesCS6B01G053900.1	Cysteine proteinase
*Qsm2-Irr.mah-6B*			
*Qgy2-Irr.mah-6B*			
*Qgns2-Irr.tab-4A*	chr4A_681683121-chr4A_684909655	TraesCS4A01G411500.1	NAD(P)H-quinone oxidoreductase subunit H
*Qgns-Wd.3P-4A*			
*Qgns2-Irr.tab-4A*			
*Qsl1-Irr.tab-4A*	chr4A_632236000-chr4A_640906743	TraesCS4A01G366600.1	F-box family protein
*Qsl-Wd.3P-2A*	chr2A_15816001-chr2A_21812312	TraesCS2A01G056900.1	Multidrug resistance protein, ABC transporter family protein
*Qsl-Wd.mia-2B*	chr2B_25477352-chr2B_26987136	TraesCS2B01G055300.1	NBS-LRR disease resistance protein-like
*Qsm-Wd.tab-2B*	chr2B_485443263-chr2B_565076231	TraesCS2B01G359800.1	Mitochondrial inner membrane protease ATP23
*Qsm2-Irr.3P-2B*	chr2B_779912793-chr2B_785208751	TraesCS2B01G596900.1	Acyl-CoA-binding domain-containing protein
*Qsm-Irr.3P-5A*	chr5A_581488651-chr5A_589302806	TraesCS5A01G383800.1	Heat shock transcription factor
*Qph2-Irr.mia-6B*	chr6B_659809861-chr6B_662562269	TraesCS6B01G384600.1	Stigma-specific protein Stig1
*Qph1-Irr.tab-4B*	chr4B_21573518-chr4B_37529724	TraesCS4B01G042200.2	Serine/threonine-protein kinase
*Qph-Wd.tab-4B*			
*Qph1-Irr.mia-6B*	chr6B_569936355-chr6B_623369219	TraesCS6B01G320500.1	F-box family protein
*Qph-Irr.tab-7A*	chr7A_82949085-chr7A_85912154	TraesCS7A01G130300.1	Protein DETOXIFICATION
*Qph1-Wd.mia-7A*	chr7A_13905540-chr7A_19958972	TraesCS7A01G036200.1	60 kDa chaperonin
*Qph1-Wd.3P-7A*			
*Qph-Wd.mah-7D*	chr7D_451622823-chr7D_554279150	TraesCS7D01G375100.1	Phosphatidylcholine:diacylglycerol cholinephosphotransferase 1
*Qbio-Wd.3P-5A*	chr5A_549192141-chr5A_555596100	TraesCS5A01G349500.1	Plant regulator RWP-RK family protein
*Qbio-Wd.mah-5A*			
*Qhi2-Irr.mia-2A*	chr2A_2810448-chr2A_13880947	TraesCS2A01G015400.1	Cytochrome P450
*Qhi2-Irr.tab-1B*	chr1B_402043386-chr1B_516951252	TraesCS1B01G286300.1	Cytochrome P450
*Qhi1-Wd.mah-3B*	chr3B_122826152-chr3B_190473903	TraesCS3B01G156900.1	B-box zinc finger family protein
*Qhi2-Wd.mah-3B*	chr3B_411269889-chr3B_452490772	TraesCS3B01G264100.1	Protein CHUP1

^a^: chromosome name_chromosome position in bp

## Supplementary Materials

The following supporting information can be downloaded at: https://www.mdpi.com/article/10.3390/plants11192533/s1, Table S1: Phenotypic performance for grain yield (GY), Plant height (PH), Number of grain per spike (GNS), 1000 kernel weight (TKW), Spike length (SL), Number of spike per m2 (SM), Biomass (BIO) and Harvest index (HI) for recombinant inbred lines and their parents across all environments under irrigated and water deficit conditions; Table S2: Coincidences of significant QTLs in this study with QTL regions reported previously; Figure S1: Coincidences of significant QTLs in this study with QTL regions reported previously. Each chromosome is represented by physical positions Mb (on the left) and identified QTLs (on the right). QTL names indicate the trait, trail (water condition+ environment) and chromosome name along with different colors: GY (red), PH (green), SM (yellow), TKW (pink), GNS (blue), SL (light blue), BIO (black), HI (brown). Identified QTLs under irrigated and water deficit conditions are represented by solid filled and stripes filled box, respectively. QTL regions reported by previous studies are shown by non-filled black box. KN: kernel number, KW: kernel width, DM: Dry mass accumulation, DSI: drought susceptibility index, NSS: number of spikelet per spike, SSN: Sterile spikelet number, CL: Coleoptile length, DTH: days to heading, DTA: days to anthesis, ShL: Shoot length, CID: carbon isotope discrimination, SG: stay green, NDVI: normalized differential vegetative index, ClF:Chlorophyll fluorescence.
