# Supplementary material for: High-Density Linkage Mapping of Agronomic Trait QTLs in Wheat under Water Deficit Condition using Genotyping by Sequencing (GBS)
Source: Plants (Basel). 2022 Sep 27;11(19):2533. doi: 10.3390/plants11192533 (PMC9571144; doi:10.3390/plants11192533)
Supplement: Supplementary file 1 [file plants-11-02533-s001.zip › plants-1863299-supplementary.pdf]

Supplementary data

Table S1. Phenotypic performance for grain yield (GY), Plant height (PH), Number of grain per spike (GNS), 1000 kernel weight (TKW), Spike length (SL), Number of spike per m^2 (SM), Biomass (BIO) and Harvest index (HI) for recombinant inbred lines and their parents across all environments under irrigated and water deficit conditions.

Trait	Location	Water condition	# 49	Yecora Rojo	RIL Population	
					Mean	Range
GY	Mahabad	Irrigated	237.86	208.02	228.26	148.44–324.21
		Water deficit	187.41	154.86	140.16	63.15–214.38
	Miandoab	Irrigated	266.23	213.13	235.39	159.74–337.95
		Water deficit	205.43	163.98	147.49	73.2–217.56
	Tabriz	Irrigated	186.05	211.03	199.78	75.58–333.77
		Water deficit	145.56	166.51	137.08	59.22–218.63
PH	Mahabad	Irrigated	64.88	48.30	58.71	37.6–82.25
		Water deficit	51.84	38.73	47.44	32.85–66.03
	Miandoab	Irrigated	68.78	46.06	60.06	37.83–82.47
		Water deficit	57.50	39.71	48.76	34.2–66.47
	Tabriz	Irrigated	74.48	43.66	58.01	39.9–78.22
		Water deficit	60.61	41.19	52.27	35.77–74.32
NGS	Mahabad	Irrigated	27.00	21.45	20.75	15.5–26.42
		Water deficit	18.10	17.80	15.74	10.7–20.8
	Miandoab	Irrigated	28.55	23.53	21.78	16.75–28.2
		Water deficit	18.35	18.25	16.86	12.1–21.13
	Tabriz	Irrigated	29.02	24.78	23.36	13.05–34.02
		Water deficit	18.49	19.88	19.21	11.22–27.05
TKW	Mahabad	Irrigated	58.11	59.09	55.71	36.02–74.88
		Water deficit	51.73	49.53	47.70	35.25–57.81
	Miandoab	Irrigated	57.70	61.17	57.27	32.6–76.05
		Water deficit	49.35	45.46	48.80	30.81–61.47
	Tabriz	Irrigated	32.50	27.94	30.39	20.87–38.85
		Water deficit	27.58	25.11	27.69	18.9–33.97
SL	Mahabad	Irrigated	8.49	7.23	7.70	6.46–8.9
		Water deficit	7.43	6.83	6.57	5.42–7.73
	Miandoab	Irrigated	8.26	7.26	8.01	6.77–-9.2
		Water deficit	7.11	6.59	6.73	5.7–7.8
	Tabriz	Irrigated	7.45	7.39	6.93	5.32–8.63
		Water deficit	6.50	6.48	6.40	4.94–8.28
SM	Mahabad	Irrigated	292.00	221.00	264.80	191–342
		Water deficit	249.00	192.00	211.04	128–295
	Miandoab	Irrigated	286.00	196.00	257.56	184–329
		Water deficit	232.00	167.00	209.65	150–275
	Tabriz	Irrigated	357.88	393.60	415.42	235.29–567.21
		Water deficit	323.64	309.95	323.98	181.89–436.47
BIO	Mahabad	Irrigated	483.86	413.54	468.98	365.31–622.76
		Water deficit	329.15	299.05	337.42	221.81–510.7
	Miandoab	Irrigated	441.76	385.16	483.80	343.5–617.5
		Water deficit	364.46	317.92	350.50	225.29-436.84

	Tabriz	Irrigated	710.44	609.69	618.57	378.62–868.36
		Water deficit	560.58	448.57	458.66	286.737–638.93
HI	Mahabad	Irrigated	49.20	50.87	49.18	32.83–65.68
		Water deficit	55.97	51.88	42.14	24.43–62.82
	Miandoab	Irrigated	60.45	56.62	50.11	30.63–72.58
		Water deficit	56.31	51.91	43.23	22.28–62.52
	Tabriz	Irrigated	26.19	35.20	32.20	18.8–40.77
		Water deficit	26.22	36.77	29.75	17.89–37.56

Table S2. Coincidences of significant QTLs in this study with QTL regions reported previously.

Chromosome	Trait	Physical interval	QTL Reported in the same interval
1B	TKW	639043667-646178536	TKW [1], GY [2], GL [3]
2A	GY, SL	20237446-33006222	PH [4–6], GY [5]
2A	HI	2810448-13880947	KN [7]
2B	PH, SM	770220812-780595416	PH [6]
2B	SM	493650087-548651192	GY, PH [8,9]
3A	GNS, SL	695611958-707999079	GY [2]
3B	SL	37637136- 44847766	SL [10]
3B	HI	122826152-190473903	KW [11]
3B	HI	411269889-452490772	DM [12]
3D	PH, HI	453986741-459223201	HI [2]
4A	GY, PH, BIO	612444080-621401013	GY [13–15], PH [16,17], DSI [14], TKW [18,19], NSS [19], SSN [17]
4A	GNS, SL	632236000-684909655	GNS [20], PH [9], TKW [20], KW [9]
4B	PH, TKW	21573518-37529724	PH [10,21], SL [22,3], GY and NGS [20]
4D	PH	112074829-342791800	PH [23,24], GY [25], CL [26], BIO [27], TKW [28]
5A	SM	581488651-589302806	PH [6], DTH and DTA [29]
5B	TKW	360428982- 445174230	TKW [27,20], GY [30], CHl-10 [20], ShL [31]
6B	TKW,PH	659589376-676681142	TKW [32], SM [25], NDVI [29]
7A	PH	15533539-28556096	BIO/CID/SG [33]
7B	PH	648784533-657872523	PH [6]
7B	TKW	693025970-703983881	TKW [1], PH [34], GNS and NSS [19]
7D	GNS, SL	89311202-105089647	GY [25], GNS [10,19], PH [6], TKW [35,1]
7D	PH	451622823-554279150	ClF [36]

GY: grain yield, TKW: 1000 kernel weight, PH: plant height, HI: harvest index, GNS: grain number per spike, SM: spike number per m2, SL: spike length, BIO: biomass, GL: grain length, KN: kernel number, KW: kernel width, DM: Dry mass accumulation, DSI: drought susceptibility index, NSS: number of spikelet per spike, SSN: Sterile spikelet number, CL: Coleoptile length, DTH: days to heading, DTA: days to anthesis, ShL: Shoot length, CID: carbon isotope discrimination, SG: stay green, NDVI: normalized differential vegetative index, ClF:Chlorophyll fluorescence.

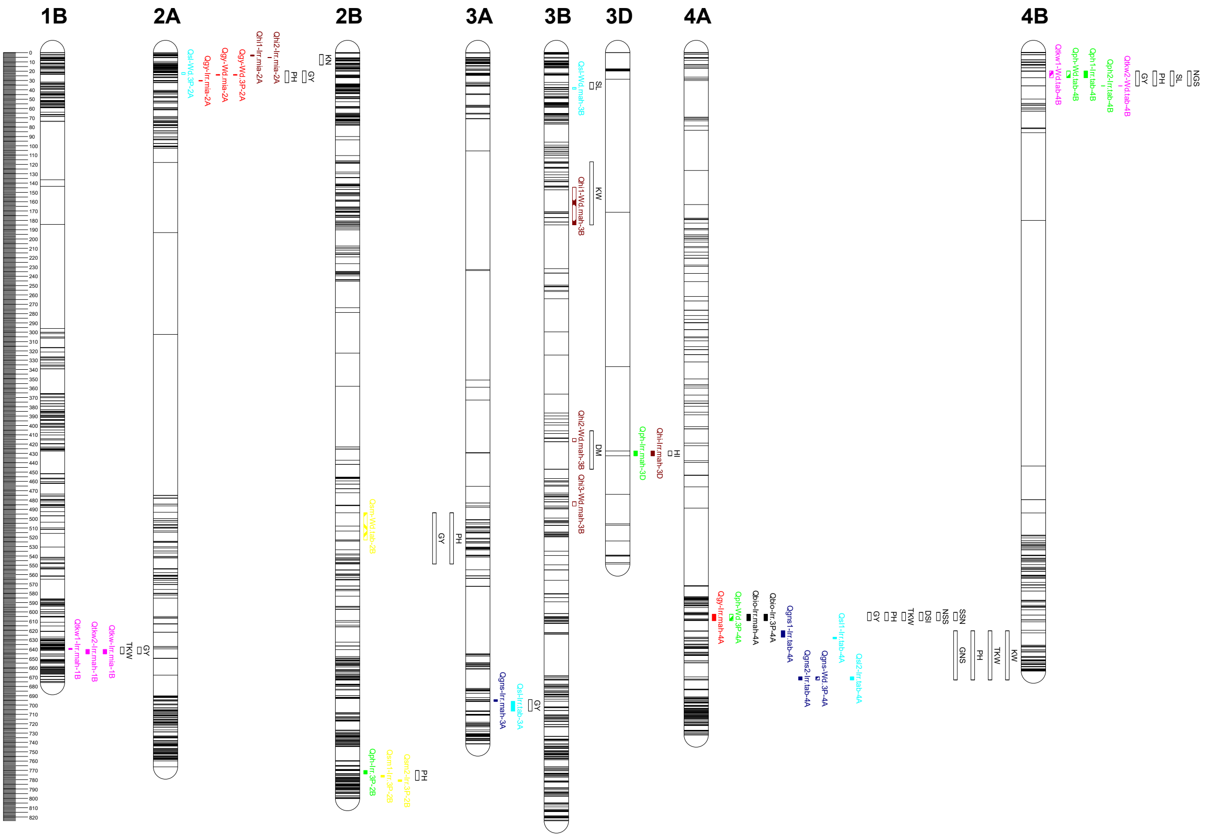

Figure S1. Coincidences of significant QTLs in this study with QTL regions reported previously. Each chromosome is represented by physical positions Mb (on the left) and identified QTLs (on the right). QTL names indicate the trait, trail (water condition+ environment) and chromosome name along with different colors: GY (red), PH (green), SM (yellow), TKW (pink), GNS (blue), SL (light blue), BIO (black), HI (brown). Identified QTLs under irrigated and water deficit conditions are represented by solid filled and stripes filled box, respectively. QTL regions reported by previous studies are shown by non-filled black box. KN: kernel number, KW: kernel width, DM: Dry mass accumulation, DSI: drought susceptibility index, NSS: number of spikelet per spike, SSN: Sterile spikelet number, CL: Coleoptile length, DTH: days to heading, DTA: days to anthesis, ShL: Shoot length, CID: carbon isotope discrimination, SG: stay green, NDVI: normalized differential vegetative index, ClF:Chlorophyll fluorescence.

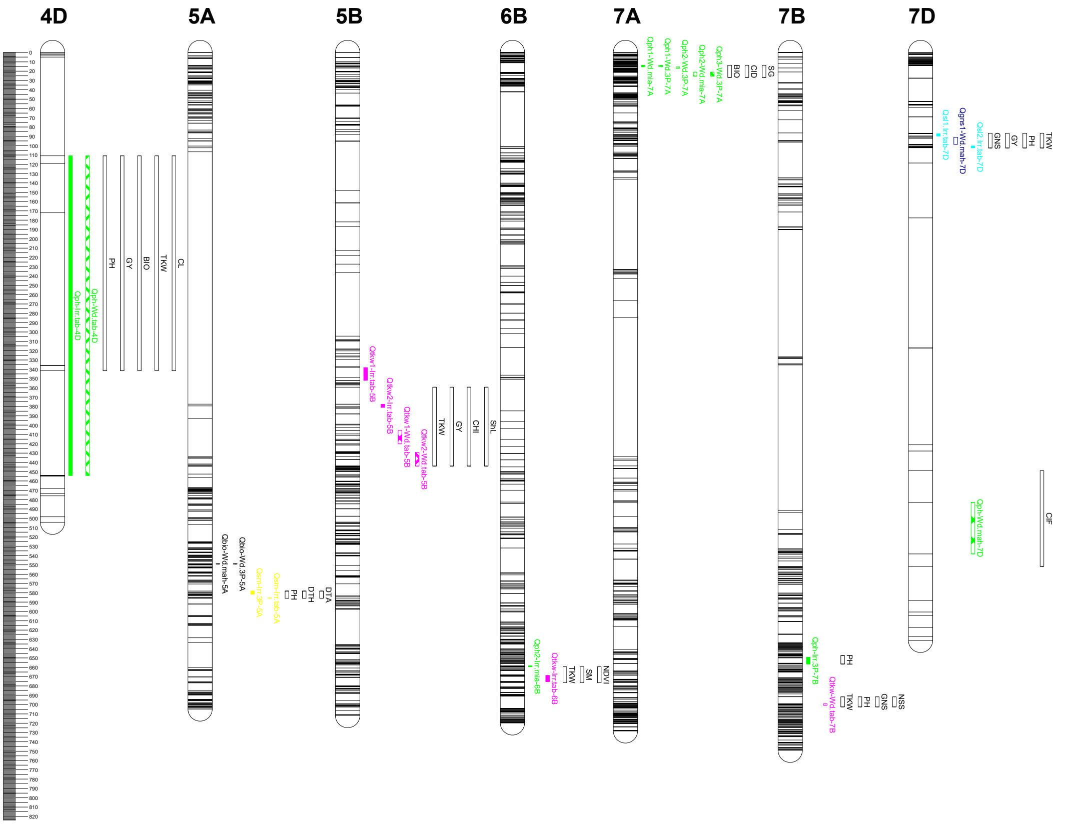

Figure S1. Continued.

References

1. Zanke, C.D.; Ling, J.; Plieske, J.; Kollers, S.; Ebmeyer, E.; Korzun, V.; Argillier, O.; Stiewe, G.; Hinze, M.; Neumann, F.; et al. Analysis of Main Effect QTL for Thousand Grain Weight in European Winter Wheat (Triticum Aestivum L.) by Genome-Wide Association Mapping. *Front. Plant Sci.* **2015**, *6*, doi:10.3389/fpls.2015.00644.
2. Bhatta, M.; Morgounov, A.; Belamkar, V.; Baenziger, P. Genome-Wide Association Study Reveals Novel Genomic Regions for Grain Yield and Yield-Related Traits in Drought-Stressed Synthetic Hexaploid Wheat. *Int. J. Mol. Sci.* **2018**, *19*, 3011, doi:10.3390/ijms19103011.
3. Pang, Y.; Liu, C.; Wang, D.; St. Amand, P.; Bernardo, A.; Li, W.; He, F.; Li, L.; Wang, L.; Yuan, X.; et al. High-Resolution Genome-Wide Association Study Identifies Genomic Regions and Candidate Genes for Important Agronomic Traits in Wheat. *Mol. Plant* **2020**, *13*, 1311–1327, doi:10.1016/j.molp.2020.07.008.
4. Griffiths, S.; Simmonds, J.; Leverington, M.; Wang, Y.; Fish, L.; Sayers, L.; Alibert, L.; Orford, S.; Wingen, L.; Snape, J. Meta-QTL Analysis of the Genetic Control of Crop Height in Elite European Winter Wheat Germplasm. *Mol. Breed.* **2012**, *29*, 159–171, doi:10.1007/s11032-010-9534-x.
5. Juliana, P.; Poland, J.; Huerta-Espino, J.; Shrestha, S.; Crossa, J.; Crespo-Herrera, L.; Toledo, F.H.; Govindan, V.; Mondal, S.; Kumar, U.; et al. Improving Grain Yield, Stress Resilience and Quality of Bread Wheat Using Large-Scale Genomics. *Nat. Genet.* **2019**, *51*, 1530–1539, doi:10.1038/s41588-019-0496-6.
6. Zanke, C.D.; Ling, J.; Plieske, J.; Kollers, S.; Ebmeyer, E.; Korzun, V.; Argillier, O.; Stiewe, G.; Hinze, M.; Neumann, K.; et al. Whole Genome Association Mapping of Plant Height in Winter Wheat (Triticum Aestivum L.). *PloS One* **2014**, *9*, e113287, doi:10.1371/journal.pone.0113287.

7. Mason, R.E.; Mondal, S.; Beecher, F.W.; Pacheco, A.; Jampala, B.; Ibrahim, A.M.H.; Hays, D.B. QTL Associated with Heat Susceptibility Index in Wheat (Triticum Aestivum L.) under Short-Term Reproductive Stage Heat Stress. *Euphytica* **2010**, *174*, 423–436, doi:10.1007/s10681-010-0151-x.
8. Mathews, K.L.; Malosetti, M.; Chapman, S.; McIntyre, L.; Reynolds, M.; Shorter, R.; van Eeuwijk, F. Multi-Environment QTL Mixed Models for Drought Stress Adaptation in Wheat. *Theor. Appl. Genet.* **2008**, *117*, 1077–1091, doi:10.1007/s00122-008-0846-8.
9. McIntyre, C.L.; Mathews, K.L.; Rattey, A.; Chapman, S.C.; Drenth, J.; Ghaderi, M.; Reynolds, M.; Shorter, R. Molecular Detection of Genomic Regions Associated with Grain Yield and Yield-Related Components in an Elite Bread Wheat Cross Evaluated under Irrigated and Rainfed Conditions. *Theor. Appl. Genet.* **2010**, *120*, 527–541, doi:10.1007/s00122-009-1173-4.
10. Li, F.; Wen, W.; He, Z.; Liu, J.; Jin, H.; Cao, S.; Geng, H.; Yan, J.; Zhang, P.; Wan, Y.; et al. Genome-Wide Linkage Mapping of Yield-Related Traits in Three Chinese Bread Wheat Populations Using High-Density SNP Markers. *Theor. Appl. Genet.* **2018**, *131*, 1903–1924, doi:10.1007/s00122-018-3122-6.
11. Chen, G.; Zhang, H.; Deng, Z.; Wu, R.; Li, D.; Wang, M.; Tian, J. Genome-Wide Association Study for Kernel Weight-Related Traits Using SNPs in a Chinese Winter Wheat Population. *Euphytica* **2016**, *212*, 173–185, doi:10.1007/s10681-016-1750-y.
12. Liang, Y.; Zhang, K.; Zhao, L.; Liu, B.; Meng, Q.; Tian, J.; Zhao, S. Identification of Chromosome Regions Conferring Dry Matter Accumulation and Photosynthesis in Wheat (Triticum Aestivum L.). *Euphytica* **2010**, *171*, 145–156, doi:10.1007/s10681-009-0024-3.
13. Kirigwi, F.M.; Van Ginkel, M.; Brown-Guedira, G.; Gill, B.S.; Paulsen, G.M.; Fritz, A.K. Markers Associated with a QTL for Grain Yield in Wheat under Drought. *Mol. Breed.* **2007**, *20*, 401–413, doi:10.1007/s11032-007-9100-3.
14. Shukla, S.; Singh, K.; Patil, R.V.; Kadam, S.; Bharti, S.; Prasad, P.; Singh, N.K.; Khanna-Chopra, R. Genomic Regions Associated with Grain Yield under Drought Stress in Wheat (Triticum Aestivum L.). *Euphytica* **2015**, *203*, 449–467, doi:10.1007/s10681-014-1314-y.
15. Li, C.; Bai, G.; Carver, B.F.; Chao, S.; Wang, Z. Single Nucleotide Polymorphism Markers Linked to QTL for Wheat Yield Traits. *Euphytica* **2015**, *206*, 89–101, doi:10.1007/s10681-015-1475-3.
16. Isham, K.; Wang, R.; Zhao, W.; Wheeler, J.; Klassen, N.; Akhunov, E.; Chen, J. QTL Mapping for Grain Yield and Three Yield Components in a Population Derived from Two High-Yielding Spring Wheat Cultivars. *TAG Theor. Appl. Genet. Theor. Angew. Genet.* **2021**, *134*, 2079–2095, doi:10.1007/s00122-021-03806-1.
17. Liu, G.; Jia, L.; Lu, L.; Qin, D.; Zhang, J.; Guan, P.; Ni, Z.; Yao, Y.; Sun, Q.; Peng, H. Mapping QTLs of Yield-Related Traits Using RIL Population Derived from Common Wheat and Tibetan Semi-Wild Wheat. *Theor. Appl. Genet.* **2014**, *127*, 2415–2432, doi:10.1007/s00122-014-2387-7.
18. Bhatta, M.; Morgounov, A.; Belamkar, V.; Baenziger, P.S. Genome-Wide Association Study Reveals Novel Genomic Regions for Grain Yield and Yield-Related Traits in Drought-Stressed Synthetic Hexaploid Wheat. *Int. J. Mol. Sci.* **2018**, *19*, E3011, doi:10.3390/ijms19103011.
19. Wu, X.; Chang, X.; Jing, R. Genetic Insight into Yield-Associated Traits of Wheat Grown in Multiple Rain-Fed Environments. *PloS One* **2012**, *7*, e31249, doi:10.1371/journal.pone.0031249.

20. Gao, F.; Wen, W.; Liu, J.; Rasheed, A.; Yin, G.; Xia, X.; Wu, X.; He, Z. Genome-Wide Linkage Mapping of QTL for Yield Components, Plant Height and Yield-Related Physiological Traits in the Chinese Wheat Cross Zhou 8425B/Chinese Spring. *Front. Plant Sci.* **2015**, *6*, doi:10.3389/fpls.2015.01099.
21. Vitale, P.; Fania, F.; Esposito, S.; Pecorella, I.; Pecchioni, N.; Palombieri, S.; Sestili, F.; Lafiandra, D.; Taranto, F.; De Vita, P. QTL Analysis of Five Morpho-Physiological Traits in Bread Wheat Using Two Mapping Populations Derived from Common Parents. *Genes* **2021**, *12*, 604, doi:10.3390/genes12040604.
22. Zhai, H.; Feng, Z.; Li, J.; Liu, X.; Xiao, S.; Ni, Z.; Sun, Q. QTL Analysis of Spike Morphological Traits and Plant Height in Winter Wheat (Triticum Aestivum L.) Using a High-Density SNP and SSR-Based Linkage Map. *Front. Plant Sci.* **2016**, *7*.
23. Zhang, K.; Tian, J.; Zhao, L.; Wang, S. Mapping QTLs with Epistatic Effects and QTL × Environment Interactions for Plant Height Using a Doubled Haploid Population in Cultivated Wheat. *J. Genet. Genomics* **2008**, *35*, 119–127, doi:10.1016/S1673-8527(08)60017-X.
24. Sun, C.; Zhang, F.; Yan, X.; Zhang, X.; Dong, Z.; Cui, D.; Chen, F. Genome-Wide Association Study for 13 Agronomic Traits Reveals Distribution of Superior Alleles in Bread Wheat from the Yellow and Huai Valley of China. *Plant Biotechnol. J.* **2017**, *15*, 953–969, doi:10.1111/pbi.12690.
25. Yang, Y.; Dhakal, S.; Chu, C.; Wang, S.; Xue, Q.; Rudd, J.C.; Ibrahim, A.M.H.; Jessup, K.; Baker, J.; Fuentealba, M.P.; et al. Genome Wide Identification of QTL Associated with Yield and Yield Components in Two Popular Wheat Cultivars TAM 111 and TAM 112. *PLoS ONE* **2020**, *15*, e0237293, doi:10.1371/journal.pone.0237293.
26. Rebetzke, G.J.; Verbyla, A.P.; Verbyla, K.L.; Morell, M.K.; Cavanagh, C.R. Use of a Large Multiparent Wheat Mapping Population in Genomic Dissection of Coleoptile and Seedling Growth. *Plant Biotechnol. J.* **2014**, *12*, 219–230, doi:10.1111/pbi.12130.
27. Ain, Q.; Rasheed, A.; Anwar, A.; Mahmood, T.; Mahmood, T.; Imtiaz, M.; He, Z.; Xia, X.; Quraishi, U. Genome-Wide Association for Grain Yield under Rainfed Conditions in Historical Wheat Cultivars from Pakistan. *Front. Plant Sci.* **2015**, *6*.
28. Zhang, H.; Chen, J.; Li, R.; Deng, Z.; Zhang, K.; Liu, B.; Tian, J. Conditional QTL Mapping of Three Yield Components in Common Wheat (Triticum Aestivum L.). *Crop J.* **2016**, *4*, 220–228, doi:10.1016/j.cj.2016.01.007.
29. Sukumaran, S.; Dreisigacker, S.; Lopes, M.; Chavez, P.; Reynolds, M.P. Genome-Wide Association Study for Grain Yield and Related Traits in an Elite Spring Wheat Population Grown in Temperate Irrigated Environments. *TAG Theor. Appl. Genet. Theor. Angew. Genet.* **2015**, *128*, 353–363, doi:10.1007/s00122-014-2435-3.
30. Perez-Lara, E.; Semagn, K.; Chen, H.; Iqbal, M.; N'Diaye, A.; Kamran, A.; Navabi, A.; Pozniak, C.; Spaner, D. QTLs Associated with Agronomic Traits in the Cutler × AC Barrie Spring Wheat Mapping Population Using Single Nucleotide Polymorphic Markers. *PLOS ONE* **2016**, *11*, e0160623, doi:10.1371/journal.pone.0160623.
31. Landjeva, S.; Neumann, K.; Lohwasser, U.; Börner, A. Molecular Mapping of Genomic Regions Associated with Wheat Seedling Growth under Osmotic Stress. *Biol. Plant.* **2008**, *52*, 259–266, doi:10.1007/s10535-008-0056-x.

32. Gahlaut, V.; Jaiswal, V.; Tyagi, B.S.; Singh, G.; Sareen, S.; Balyan, H.S.; Gupta, P.K. QTL Mapping for Nine Drought-Responsive Agronomic Traits in Bread Wheat under Irrigated and Rain-Fed Environments. *PLOS ONE* **2017**, *12*, e0182857, doi:10.1371/journal.pone.0182857.
33. Acuña-Galindo, M.A.; Mason, R.E.; Subramanian, N.K.; Hays, D.B. Meta-Analysis of Wheat QTL Regions Associated with Adaptation to Drought and Heat Stress. *Crop Sci.* **2015**, *55*, 477–492, doi:10.2135/cropsci2013.11.0793.
34. Wang, S.-X.; Zhu, Y.-L.; Zhang, D.-X.; Shao, H.; Liu, P.; Hu, J.-B.; Zhang, H.; Zhang, H.-P.; Chang, C.; Lu, J.; et al. Genome-Wide Association Study for Grain Yield and Related Traits in Elite Wheat Varieties and Advanced Lines Using SNP Markers. *PLOS ONE* **2017**, *12*, e0188662, doi:10.1371/journal.pone.0188662.
35. Huang, X.Q.; Kempf, H.; Ganal, M.W.; Röder, M.S. Advanced Backcross QTL Analysis in Progenies Derived from a Cross between a German Elite Winter Wheat Variety and a Synthetic Wheat (Triticum Aestivum L.). *TAG Theor. Appl. Genet. Theor. Angew. Genet.* **2004**, *109*, 933–943, doi:10.1007/s00122-004-1708-7.
36. Yang, D.-L.; Jing, R.-L.; Chang, X.-P.; Li, W. Quantitative Trait Loci Mapping for Chlorophyll Fluorescence and Associated Traits in Wheat (Triticum Aestivum). *J. Integr. Plant Biol.* **2007**, *49*, 646–654, doi:10.1111/j.1744-7909.2007.00443.x.
